# Lazarus ecology: Recovering the distribution and migratory patterns of the extinct Carolina parakeet

**DOI:** 10.1002/ece3.3135

**Published:** 2017-06-12

**Authors:** Kevin R. Burgio, Colin J. Carlson, Morgan W. Tingley

**Affiliations:** ^1^ Department of Ecology and Evolutionary Biology University of Connecticut Storrs CT USA; ^2^ Department of Environmental Science, Policy and Management University of California Berkeley CA USA

**Keywords:** Carolina parakeet, *Conuropsis carolinensis*, distribution modeling, extinction, natural history, niche comparison, seasonal movements, species distribution models

## Abstract

The study of the ecology and natural history of species has traditionally ceased when a species goes extinct, despite the benefit to current and future generations of potential findings. We used the extinct Carolina parakeet as a case study to develop a framework investigating the distributional limits, subspecific variation, and migratory habits of this species as a means to recover important information about recently extinct species. We united historical accounts with museum collections to develop an exhaustive, comprehensive database of every known occurrence of this once iconic species. With these data, we combined species distribution models and ordinal niche comparisons to confront multiple conjectured hypotheses about the parakeet's ecology with empirical data on where and when this species occurred. Our results demonstrate that the Carolina parakeet's range was likely much smaller than previously believed, that the eastern and western subspecies occupied different climatic niches with broad geographical separation, and that the western subspecies was likely a seasonal migrant while the eastern subspecies was not. This study highlights the novelty and importance of collecting occurrence data from published observations on extinct species, providing a starting point for future investigations of the factors that drove the Carolina parakeet to extinction. Moreover, the recovery of lost autecological knowledge could benefit the conservation of other parrot species currently in decline and would be crucial to the success of potential de‐extinction efforts for the Carolina parakeet.

## 1 INTRODUCTION

The sixth mass extinction has dominated ecological research in the last decade, but by and large, recently extinct species are a dead end for natural historical inquiry beyond paleontological research. Efforts to recover the natural history of recently extinct species have been primarily restricted to Pleistocene megafauna (Donlan et al., 2006), although such comparisons have largely focused on the ecological suitability of extant surrogates (Richmond, McEntee, Hijmans, & Brashares, 2010). Nonetheless, the extent of basic biological and ecological knowledge of recently extinct species varies greatly. For some species, an ecological signature remaining in extant species may be enough to infer an extinct role, as with the antiherbivore plant defenses that highlight the lost function of elephant birds (Aepyornithidae) in Madagascar and moas (Dinornithidae) in New Zealand (Bond & Silander, 2007). But the majority of extinctions are poorly documented, and conjectures on the ecological role of extinct species have led to misinterpretations, as echoed in the multidecade controversy over, and ultimate rejection of, Temple's hypothesis (Temple, 1977) that the extinction of the dodo was the cause of the decline of the calvaria tree (*Sideroxylon grandiflorum* A.DC.; see Hershey, 2004).

Recent advances in ecological modeling have made the recovery of extinct species’ biology more plausible and less perilous. Various new methods provide researchers a more formal approach to testing hypotheses, rather than relying on conjecture based on anecdotal observations. The potential for rediscovering our lost natural history has been on the minds of ecologists with the recent centennial anniversary of the death of the last captive passenger pigeon (*Ectopistes migratorius* Linnaeus) in 1914, and the controversial “resighting” of the ivory‐billed woodpecker (*Campephilus principalis* Linnaeus, Fitzpatrick et al., 2005 but see Sibley, Bevier, Patten, & Elphick, 2006). Indeed, much recent research has focused on these recently extinct, iconic North American birds (e.g., Gotelli, Chao, Colwell, Hwang, & Graves, 2012; Hung et al., 2014; Stanton, 2014), but this research largely focuses on attempts to determine exact extinction dates and immediate causes of extinction. By contrast, another iconic, extinct, North American bird, the Carolina parakeet (*Conuropsis carolinensis* Linnaeus), has received relatively less attention, especially over the past 30 years.

The most recent estimated extinction date of the Carolina parakeet is 1915 (Elphick, Roberts, & Reed, 2010), with the last captive individual dying in 1918 in the Cincinnati Zoo (curiously, in the same zoo the last captive passenger pigeon died 4 years earlier; Laycock, 1969), although it is likely the species persisted until the 1930s or beyond (Snyder, 2004). By the time the Carolina parakeet was subjected to any sustained attention by ornithologists, it was already deemed too late to learn much about their biology, so most pre‐extinction research focused on preserving specimens for museums (Snyder, 2004). Natural history accounts of the Carolina parakeet come primarily from early American ornithologists, such as Alexander Wilson and John J. Audubon. Although fairly common during the time of Wilson and Audubon, their descriptions are rife with speculation and second‐hand reports, which increase the uncertainty about even the most basic understanding of this species’ biology. Other than the extensive historical research done by McKinley (e.g., McKinley, 1960, 1977) and Snyder (e.g., Snyder, 2004), little research has been conducted on the Carolina parakeet since its extinction and that research has highlighted our lack of even basic natural history knowledge of the species.

Understanding the ecological impact of species—whether extinct or extant—on their environments, is a fundamental component of community and restoration ecology. Thus, despite being deceased, many key questions remain regarding the biology and ecological role of the Carolina parakeet. Specifically, the ecological validity of the two named subspecies (*C. c. ludovicianus* and *C. c. carolinensis*), determined largely by differences in color and size, remains equivocal (Snyder & Russell, 2002). Moreover, the Carolina parakeet's historic range is poorly documented, due primarily to a lack of formal observation. Central to the question of distribution is how a member of a tropical clade of parrots (see Kirchman, Schirtzinger, & Wright, 2012) survived, ecologically and physiologically, in a native range throughout much of eastern temperate North America. Throughout the early accounts and postextinction discussions, naturalists and ornithologists disagreed about whether or not Carolina parakeets migrated or seasonally shifted their range (for a detailed discussion, see McKinley, 1977). However, many historical accounts give conflicting information, making it difficult to determine to what extent, if at all, Carolina parakeets migrated to survive cold temperatures in the northern parts of their range.

Here, we construct a comprehensive dataset uniting and carefully georeferencing historical observations from all known accounts of the species with information contained in preserved museum specimens to (1) empirically delineate the climatic niche and range of the Carolina parakeet; (2) test for differences in the climatic associations between the two purported subspecies; and (3) assess evidence for seasonal migration through climatic niche shifts. Evaluating these questions with a novel dataset provides an opportunity to recover seemingly lost autecological information about an extinct species, and to start to understand the ecological context of the Carolina parakeet in North American temperate forest and plains ecosystems. Doing so gives us a reasonable starting point for understanding how a cosmopolitan species became extinct in a rapid decline riddled with conflicting reports of causation. Our analysis also provides a framework for recovering similar information about other lost species which may help in investigating the causes of range contraction and species extinction, and aid reintroduction efforts if extinct species are targeted for de‐extinction.

## 2 METHODS

### 2.1 Occurrence data

We collected and georeferenced locality data from Carolina parakeet specimens found in natural history collections around the world (*n* = 396; see Table S1 for list of natural history collections) and observations of Carolina parakeets published in the literature from 1564 to 1938 (*n* = 396 [sic]; see Table S2 for list of citations), using guidelines established by Chapman and Wieczorek (2006), and the software GEOLocate (Rios & Bart, 2010). Rather than using coordinates already associated with museum specimens, we chose to re‐estimate all geographical coordinates based on collection locality names to ensure consistency throughout the dataset. Given that place names and geographical extents have changed much in the past few hundred years in North America, we paid special attention to historically relevant maps and field journals of specimen collectors when selecting coordinates and measuring uncertainty for each occurrence point.

After georeferencing, we split the dataset by subspecies. We considered all occurrence points west of the Appalachian crest and west of Alabama to represent *C. c. ludovicianus* (*n* = 299) and points east of the Appalachian crest and east of Mississippi to represent *C. c. carolinensis* (*n* = 493). These broad geographical delineations are generally accepted as the range limits of the two subspecies (Ridgway, 1916; Swenk, 1934), and are consistent with the subspecies identifications listed on all 261 labeled museum specimens.

To prepare occurrence data for analysis, we first removed all duplicate sightings (i.e., sightings with more than one observation/specimen at the same location). We next removed vagrant sightings (*n* = 23) from the analysis, consistent with IUCN's definition of a species’ range (Gärdenfors, Hilton‐Taylor, Mace, & Rodríguez, 2001), which included all sightings from states where Carolina parakeets were not known to breed, and for which there are no credible records of observations during the breeding season. This rule excluded observations from the U.S. states of Colorado, Maryland, Michigan, Minnesota, New Jersey, North Dakota, Pennsylvania, and South Dakota. The removal of likely vagrants also is known to improve distributional model performance (Soley‐Guardia, Radosavljevic, Rivera, & Anderson, 2014). We also removed occurrence points from analyses if the radius of uncertainty associated with a point was greater than 5 km, as this level of uncertainty reduces the accuracy of resulting species distribution models (Graham et al., 2008). This procedure limited our combined specimen and observation dataset to a total of 330 high‐quality and unique georeferenced occurrence points across both subspecies.

To avoid overfitting models due to spatial autocorrelation, we further thinned each subspecies’ dataset using the “spThin” R package (Aiello‐Lammens, Boria, Radosavljevic, Vilela, & Anderson, 2015). We used a nearest‐neighbor distance of 9 km, which corresponds to the typical home‐range size for small to medium‐sized parrots belonging to the Carolina parakeet's subfamily Arinae (Vehrencamp, Ritter, Keever, & Bradbury, 2003), as the Carolina parakeet's home‐range size is undocumented. After thinning data, 147 unique georeferenced locations were used in the analyses (*C. c. ludovicianus n* = 99; *C. c. carolinensis n* = 48).

The extent of analysis, and therefore, selection of 1,000 background samples—“pseudo‐absences” (Merow, Smith, & Silander, 2013)—was confined to the specific set of North American ecoregions (Olson et al., 2001) where each subspecies of Carolina parakeet was observed historically. This approach allows a more meaningful assessment of each subspecies’ niche by including areas that were accessible to the species (Barve et al., 2011; Soberon & Peterson, 2005). Using extents with no biological basis (i.e., geopolitical boundaries) can artificially inflate evaluations of model fit (i.e., area under the curve, AUC), giving false confidence in the validity of the model (Jimenez‐Valverde, Lobo, & Hortal, 2008).

### 2.2 Climatic data

We derived 19 climatic variables (e.g., mean annual temperature and mean annual precipitation; see Hijmans, Cameron, Parra, Jones, & Jarvis, 2005 for variable descriptions) from a 30‐year window of 4 km resolution climate data (1895–1924) downloaded from the PRISM Climate Group (Oregon State University, http://prism.oregonstate.edu, created 4 Feb 2004) using the “dismo” package (v. 2.13.0; Hijmans, Phillips, Leathwick, & Elith, 2012) in R (R Core Team 2013). We used the 1895–1924 timeframe because it overlaps with the final period during which the Carolina parakeet was extant and avoids the climate warming trend that started around 1950 (see Stanton, 2014).

### 2.3 Subspecies niche comparison

To test for potential differences between each subspecies’ climatic niche, we divided the occurrence data by subspecies and used niche equivalency tests (Warren, Glor, & Turelli, 2008) of ordinal niche comparisons (Broennimann et al., 2012) in the R package “ecospat” (v. 1.1; Di Cola et al., 2017) to test for differentiation between climatic niches of the purported subspecies. However, some have argued that niche identity tests are likely to overpredict differences between species, suggesting the use of Warren et al.'s (2008) background test, which corrects for the environmental covariate space in the species’ available area (Peterson, 2011). We implement both analyses in the R package “ENMTools” (v. 0.1; Warren, Glor, & Turelli, 2010), using a 90% minimum training presence threshold for environmental space, applied to a PCA of the climate variable set.

### 2.4 Species distribution modeling

We used MaxEnt (Phillips, Anderson, & Schapire, 2006) in the R package “dismo” (v. 2.13.0; Hijmans et al., 2012) to generate species distribution models for each subspecies independently. As the Carolina parakeet was the only native parrot to the United States, and its biology is so poorly understood, we had no a priori expectations as to which climate variables may have been important in determining their range limits. So, rather than use all 19 bioclimatic variables available (sensu Hijmans et al., 2005), we limited our analysis to six climate variables (annual mean temperature, mean diurnal range, temperature seasonality, mean temperature of driest quarter, annual precipitation, and precipitation of the warmest quarter), as these variables have been shown to generally be the most important when building species distribution models for North American birds and are minimally correlated with one another (Barbet‐Massin & Jetz, 2014).

Once generated, the MaxEnt species distribution models were “tuned” using the R package “ENMeval” (v. 0.2.0; Muscarella et al., 2014), which uses a checkerboard cross‐validation method to compare the Akaike information criterion (AIC) of MaxEnt models under all combinations of model feature types to select the features that maximize the predictive ability of the model (Muscarella et al., 2014). We then selected the parameterizations that resulted in the model with the lowest AIC to run the final MaxEnt models (for AIC scores and parameters, see Table S3). Using the results of the tuned MaxEnt models, we generated distribution maps with a thresholded value which maximized the True Skill Statistic, which optimizes specificity and sensitivity (Liu, Berry, Dawson, & Pearson, 2005). Whereas approaches like thresholding based on kappa have received some criticism in the literature, the TSS approach is accurate independent of prevalence (Allouche, Tsoar, & Kadmon, 2006), and still offers a somewhat stricter threshold than minimum training presence‐based methods (which might be particularly sensitive to outlying points and unremoved vagrants in our 500‐year dataset).

### 2.5 Seasonal shifts

We evaluated differences between the breeding season and winter for each subspecies, separately, by first removing all data without month or season information and binning the resulting occurrence data into the “breeding period” (March–August; Snyder & Russell, 2002; *C. c. ludovicianus n* = 57 and *C. c. carolinensis n* = 33) and “winter” (all observations falling in December, January, or February; *C. c. ludovicianus n* = 35 and *C. c. carolinensis n* = 41). For these analyses, we excluded occurrence data that fell outside the breeding period and winter (i.e., part of spring or fall). We used niche equivalency tests (Warren et al., 2008) of ordinal niche comparisons (sensu Broennimann et al., 2012) run in the R package “ecospat” (v. 1.1; Di Cola et al., 2017) to evaluate differences between the climatic niche of each season for each subspecies using all 19 bioclimatic variables. Lastly, we generated MaxEnt SDMs in the R package “dismo” (v. 2.13.0; Hijmans et al., 2012), based on the parameterization resulting in the lowest AIC model in the R package “ENMEval” (v. 0.2.0; Muscarella et al., 2014) for each subspecies, using the season‐specific datasets and the same distribution modeling methods as described above (see Table S4).

## 3 RESULTS

**Figure 1:**
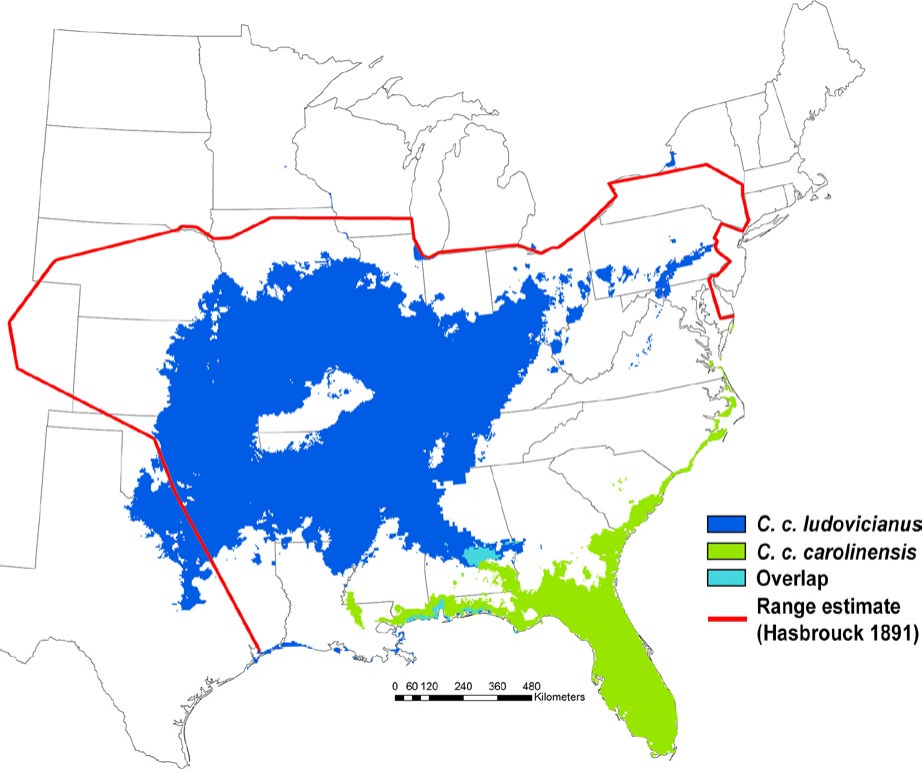
Map showing the results of the MaxEnt SDMs of *C. c. ludovicianus* (blue) and *C. c. carolinensis* (green) with areas of overlap in light green. The heavy red outline is the range boundary from the map drawn by Hasbrouck (1891). For the full probability maps and AUCs, see Figures S1 and S2

**Figure 2:**
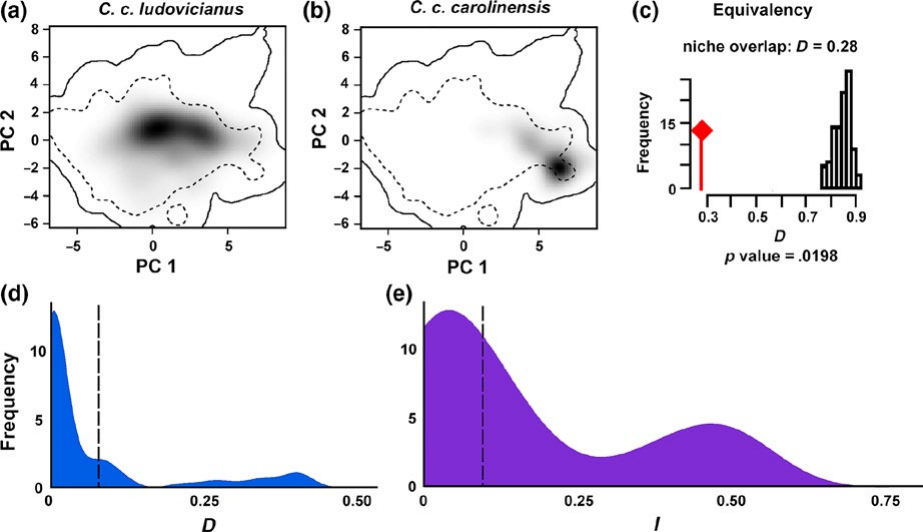
Results of the “within‐environment” PCA niche equivalency analyses between the western (a) and eastern (b) subspecies of the Carolina parakeet. The shading reflects the density of occurrences of each subspecies per cell (i.e., darker areas have a higher density), the solid line within the PCA space represents 100% of the available climate space, and the dotted lines represent 50% of the available climate space. (c) The red flag is the empirical niche overlap (*D* = 0.28) and the histograms represent the simulated overlap between the two subspecies. For the PCA correlation circle, see Figure S3. (d) Correcting for background differences between the subspecies’ accessible area, no significant difference can be found between the subspecies in Schoener's D (*p* = .267) or (e) in Warren's I (*p* = .327)—indicating that apparent differences between subspecies niches were likely attributable to geography, not autecology

Species distribution models (SDMs) indicated that the two subspecific Carolina parakeet groupings differed in climatic niche (Figures 1 and 2) with significantly little environmental overlap (Schoener's *D* = 0.28, *p* = .012; Figure 2). However, the more conservative test (Warren et al., 2008) found that once the differences in environmental background were accounted for, the subspecies’ niches were not significantly different (Schoener's *D, p* = .267, Warren's I, *p* = .327; Figure 2d,e). The two groupings additionally responded to different climate variables. For example, mean temperature of the coldest quarter was the most important climate variable contributing to the distribution of *C. c. ludovicianus* (33.9% contribution to model) while mean annual temperature was most important variable for *C. c. carolinensis* (68.4% contribution to the model). Thus, rather than creating a single spatial model for the entire species, we created two separate models, one for each subspecies (Figure 1). The AUC values for the *C. c. ludovicianus* and *C. c. carolinensis* models were 0.790 and 0.814, respectively, indicating adequate model fit (Figures S1 and S2).

**Figure 3:**
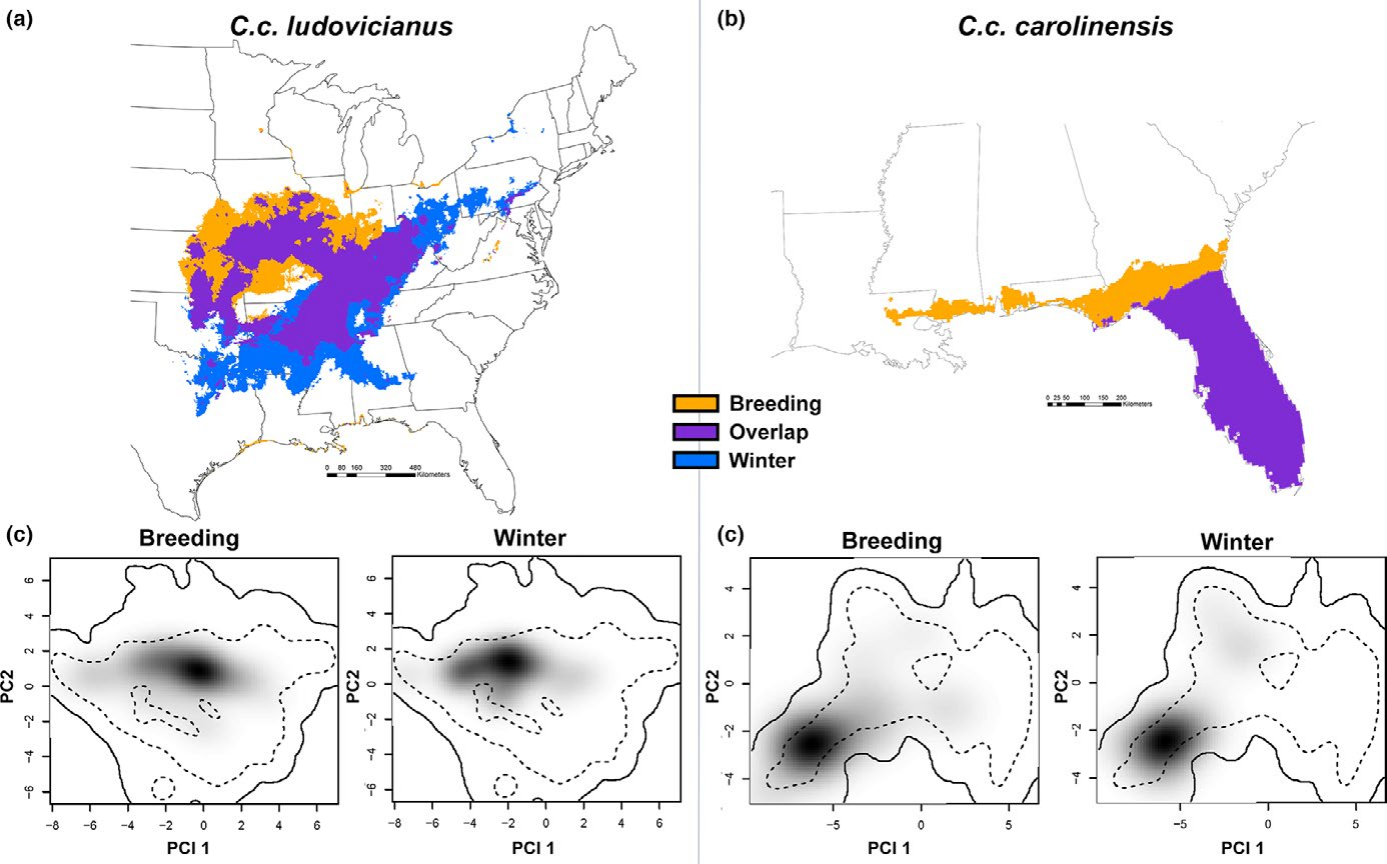
Maps show MaxEnt SDMs generated from occurrence data partitioned by “breeding” season (March through August; orange) and the winter months (December through February; blue), with areas of breeding and winter model overlap in purple for *C. c. ludovicianus* (a) and *C. c. carolinensis* (b). For full prediction maps, see Figures S6–S9. The lower panels show the results of the “within‐environment” PCA niche equivalency between the breeding and winter months for *C. c. ludovicianus* (c): *D* = 0.684, *p* = .0396); and *C. c. carolinensis* (d): *D* = 0.803, *p* = .851. In both (c) and (d), the solid lines within the PCA space represent 100% of the available climate space and the dotted lines represent 50% of the available climate space. For the PCA correlation circles and niche equivalency histograms for both *C. c. carolinensis* and *C. c. ludovicianus*, see Figures S4 and S5

We further evaluated whether each subspecies underwent seasonal migrations by testing for equivalency of climatic niches across seasons. Our results documented a significant difference between the winter and breeding season climatic niche for *C. c. ludovicianus* (*D* = 0.684, *p* = .0396; Figures 3a,c, and S4); however, there was no significant difference for *C. c. carolinensis* (*D* = 0.803, *p* = .851; Figures 3b,d, and S5). Season‐specific distribution models showed high degrees of model fit (AUC values: *C. c. ludovicianus* breeding = 0.863 and winter = 0.885, Figures 3a, S6, and S7; *C. c. carolinensis* breeding = 0.845 and winter = 0.916, Figures 3b, S8, and S9).

## 4 DISCUSSION

Our results provide strong evidence that the Carolina parakeet's range was likely much smaller than previously believed (Figure 1) and was divided across two geographically distinct ranges. Whether each subspecies had a distinct climatic niche, however, was uncertain from our analysis (Figure 2). This finding of range size, however, may help partially explain why the Carolina parakeet went extinct as quickly as it did, as populations with smaller range sizes are more vulnerable to extinction (Payne & Finnegan, 2007). This finding is parsimonious with psittacine ecology, as the previous estimate of their range size was more than 10 times larger than the average range size of all other recently extinct parrot species (Olah et al., 2016).

Comparisons of seasonal distribution models indicate that the western subspecies may have moved between breeding and winter seasons, whereas the eastern subspecies appears to have not (Figure 3). These results also suggest that the subspecific taxonomy may, in fact, be valid, despite the fairly ambiguous morphological evidence previously used to support two separate subspecies (Snyder & Russell, 2002). Although preliminary genetic work has gone as far as to place the Carolina parakeet within the subfamily Arinae in the parrot phylogeny (Kirchman et al., 2012), further genetic testing could be targeted to verify the validity of these subspecies, and to explore whether or not gene flow existed between the two subspecies in areas where they may have overlapped in the southeastern United States.

Previous range maps for this species were generated by drawing a polygon encompassing all of the most distant areas in which the Carolina parakeet had been reported (see Hasbrouck, 1891; Snyder & Russell, 2002; Figure 1). Our results suggest that the Carolina parakeet's range was much smaller than previously believed (Figure 1), including being smaller than a recently published model of the Carolina parakeet's distribution (Peers, Thornton, Majchrzak, Bastille‐Rousseau, & Murray, 2016), which used a smaller dataset that included occurrences of presumed vagrants and did not account for incorrect or highly uncertain georeferences in online databases. Moreover, there are no recorded sightings of Carolina parakeets at higher elevations in the Appalachian or Ozark Mountains, an absence reflected in the distribution models presented here (Figures 1 and 3). As the Carolina parakeet managed to live through cold winters in parts of the Midwest, the fact that they were not found in the higher elevations is perplexing. A possible explanation may be that Carolina parakeets are most frequently associated with bald cypress (*Taxodium distichum* (L.) Rich), as both an important food source and nesting tree (Snyder & Russell, 2002). Bald cypresses generally grow only at elevations <30 m above sea level (Fowells, 1965). Overall, the native range of the bald cypress (Little, 1971) overlaps almost completely with the range of *C. c. carolinensis* and the year‐round portion of the range of *C. c. ludovicianus* (Figures 1 and 3a).

Finally, our findings on seasonal migration corroborate the suspicions of McKinley (1977), who conjectured that *C. c. ludovicianus* shifted its range away from the northwest portion of its distribution in the winter. Although there are documented observations of Carolina parakeets during temperatures as low as −30°C in Nebraska (Wilson, 1811) and −32°C in southern Indiana (Wied, 1839), it is unclear whether Carolina parakeets could have survived such low temperatures for a sustained period. Our results provide ecological evidence that *C. c. ludovicianus* migrated between seasons, while the eastern subspecies, *C. c. carolinensis,* did not (Figure 3). Such a marginal migration pattern is found in other forest‐dwelling nonpasserines, such as the red‐headed woodpecker (*Melanerpes erythrocephalus* Linnaeus), which has a very similar (though slightly larger) range that shifts southeasterly out of the upper Midwest USA in the winter (Frei, Smith, Withgott, & Rodewold, 2015) depending on food availability (Smith, 1986). Insufficient data on *C. c. carolinensis* outside of Florida may contribute bias to our results that fail to support a seasonal migration within that range; however, given that there are comparable numbers of observations in both summer and winter for this subspecies, it seems unlikely that any effect of limited sampling is biased seasonally.

Seasonal migration should be considered as just one of a number of adaptations that could have helped Carolina parakeets persist in colder areas than their closest relatives, which are largely tropical in distribution (Kirchman et al., 2012). For example, Carolina parakeets roosted communally in tree cavities year‐round, and had fully feathered ceres (Snyder & Russell, 2002). Both traits may have had thermoregulatory benefits in seasonally cold climates. Whether or not the species entered torpor is unknown, but anecdotal observations of difficult‐to‐rouse individuals are strongly suggestive of this additional adaptation to cold stress (Butler, 1892; Snyder & Russell, 2002). However, as there are many observations of active Carolina parakeets during the winter, torpor would have likely been entered only briefly and facultatively (e.g., at night). Given our results, it is likely that a combination of minor seasonal shifts, gregarious roosting, and perhaps other adaptations allowed Carolina parakeets to persist in the colder parts of their range. This mix of characteristics is also found in an extant parrot species well known for surviving in cooler climates, the monk parakeet (*Myiopsitta monachus* Boddaert), which are largely sedentary but also have a fully feathered cere and roost communally throughout the year (Burgio et al., 2016). The monk parakeet now persists in multiple invasive colonies throughout the former range of the Carolina parakeet.

### 4.1 Future directions

The task conservation faces after a species’ extinction is ambiguous. As a crisis discipline, conservation's focus is generally on identifying actions to apply to species that might still be saved. But if we hope to conserve the estimated 7.9% of all species threatened with extinction in the near future from climate change (Urban, 2015), we must understand extinction as a process. Recovering the basic biology of species that were not saved is a fundamental component of the crucial, last step in understanding extinction as a process: the end, when species actually go extinct. Our study demonstrates that the loss of a species does not necessarily mean a loss of information about its natural history—information that may prove useful in uncovering the factors that led to the species’ extinction and in informing modern conservation programs focused on threatened, closely related species. This point is especially prescient with respect to parrots, as they are the most threatened avian order, with 42% of all parrot species listed as threatened or endangered by the IUCN (Marsden, Royle, & Downs, 2015).

Although our study relied on the use of ecological niche modeling, numerous other tools can be applied to posthumously investigate natural history. Stable isotope ecology provides critical insights into diet (Hilderbrand et al., 1996), metabolism (Nelson, Angerbjörn, Lidén, & Turk, 1998), and even migration (Hoppe, Koch, Carlson, & Webb, 1999). Genetic work in conjunction with morphological analyses can be used to study population structure (Mona et al., 2010) and to resolve evolutionary history and species boundaries (Avise & Nelson, 1989; Leonard et al., 2005), to clarify the identity of ambiguous specimens like eggs (Chilton & Sorenson, 2007), and even to propose hybrid species origins (Roy, Girman, Taylor, & Wayne, 1994). We advocate for the application of these methods in conjunction with spatial tools as a more formalized toolbox for recovering the biology of extinct species, and more generally, for exploring the extinction process. We suggest genetic and stable isotope work as a future direction for research on the Carolina parakeet and other recently extinct species. With new information on the basic biology emerging from this and future studies, as well as a spatiotemporal dataset lending itself to extinction‐relevant modeling, we believe it may soon be possible to reopen the “cold case” of the Carolina Parakeet's extinction, and more rigorously resolve hypotheses explaining its sudden and precipitous decline.

Finally, recovered autecological information about extinct species may have practical applications. For instance, the Carolina parakeet is considered one of the best candidates for “de‐extinction” (Seddon, Moehrenschlager, & Ewen, 2014). De‐extinction is a process in which DNA is extracted from museum specimens and used in efforts to “bring back” extinct species (Sherkow & Greely, 2013). As more and more species go extinct, conservation options become more limited, which may make such a controversial idea more appealing. Although ethical and practical issues surround this approach to conservation (see Nogués‐Bravo, Simberloff, Rahbek, & Sanders, 2016; Sandler, 2014), the de‐extinction literature is expanding rapidly. So far, much attention has focused on selecting species that are good candidates for de‐extinction (Seddon et al., 2014) and on the development of techniques required to bring back an extinct species (Church & Regis, 2012). While initial research on evaluating habitat suitability for potential de‐extinction projects has just begun (e.g., Peers et al., 2016), the best possible knowledge of the inhabited environment, realized niche, and autecology of any candidate species will be required to successfully reintroduce populations into the wild (Seddon et al., 2014), as well as fully evaluate present and future habitat suitability.

## CONFLICT OF INTEREST

None declared.
