# Supplementary material for: Lazarus ecology: Recovering the distribution and migratory patterns of the extinct Carolina parakeet
Source: Ecol Evol. 2017 Jun 12;7(14):5467–75. doi: 10.1002/ece3.3135 (PMC5528215; doi:10.1002/ece3.3135)
Supplement: Supplementary file 1 [file ECE3-7-5467-s001.docx]

**Supplemental Information**

**Table S1: Museums with specimens used in this study**

Biodiversity occurrence data published by: American Museum of Natural History; Benedictine College; California Academy of Sciences; Cambridge University; Carnegie Museum of Natural History; Chicago Academy of the Sciences; Cornell University Museum of Vertebrates; Delaware University Museum of Vertebrates; Field Museum of Natural History; Filson Natural History Museum; Forschungsinstitut und Natur-Museum Senckenberg; French National Museum of Natural History; Harvard University Museum of Comparative Zoology; Kansas City Public Library; Marseille Muséum D'histoire Naturelle; Milwaukee Public Museum; Moore Laboratory of Zoology at Occidental College; Natural History Museum, London; Natural History Museum of Los Angeles County; New Brunswick Museum; New York State Museum; Ohio State University Museum of Zoology; Philadelphia Academy of Natural Sciences; Royal Ontario Museum; San Diego Natural History Museum; Santa Barbara Museum of Natural History; University of Amsterdam Zoological Museum; University of Berlin Zoological Museum; University of California at Berkeley Museum of Vertebrate Zoology; University of California at Los Angeles Department of Ecology and Evolutionary Biology; University of Kansas Museum of Natural History; University of Michigan Museum of Zoology; U.S. National Museum of Natural History; and the Yale University Peabody Museum (Accessed through GBIF Data Portal; data.gbif.org).

**Table S2: Citations for all observations georeferenced in occurrence dataset**

Abert, J.W. 1882. List of birds observed on a march from Ft. Leavenworth to Santa Fe in 1846 and 1847. Journal of the Cincinnati Society of Natural History 5:57-59.

Abert, J.W. 1846. Journal of Lieut. J. W. Abert from Bent's fort to St. Louis, in 1845. U.S. Senate Doc. 438, Vol. 8, 29th Congress, 1st Sess.

Allen, J.A. 1871. On the mammals and winter birds of east Florida. Florida Bulletin of the Museum of Comparative Zoology 2:161-450.

Allen, W.T. 1959. A Massaachusetts traveler on the Florida frontier (A.W. Thompson, ed). Florida History Quarterly 38:129-141.

Alexander, J.E. 1833. Transatlantic sketches. Key & Biddle, Philadelphia.

Alston, J.M. 1953. Rice Planter and Sportsman; the Recollections of J. Motte Alston, 1821-1909. University South Carolina Press, Columbia.

Anderson, R.M. 1907. The birds of Iowa. Proceedings of the Davenport Academy of Sciences 11:125-147.

Anderson, T.G. 1882. Personal Narrative of Capt. Thomas G. Anderson. Wisconsin History Collection 9:137-206.

Anonymous. 1967. Flocks of parakeets once feasted on Florida berries. Palm Beach Record.

Ashe, T. 1808. Travels in America, performed in 1806. E.M. Blunt, London.

Atherton, L.E. 1944. Life, labor, and society in Boone County, Missouri, as revealed in the correspondence of an immigrant slave-owning family from North Carolina. Monthly History Review 38:277-304, 408-429.

Atwater, C. 1838. A history of the state of Ohio, natural and civil. Glezan and Shepard, Cincinnati, OH.

Audubon, J.J. 1831. Ornithological biography. Volume 1. Edinburgh.

Audubon, J.J. 1929. Journal of John James Audubon made during his trip to New Orleans in 1820-1821. Club of Odd Volumes, Cambridge.

Audubon, J.J. 1930. Letters of John James Audubon 1826-1840. Club of Odd Volumes, Boston.

Audubon, J.J. 1942. Journey up the Mississippi. Journal of the Illinois State History Society 35:148-173.

Audubon M. 1897. Audubon and his journals. Scribners, New York, NY.

Audubon, M. and E. Coues. 1898. Audubon and his journals with zoological and other notes by Coues. John C. Nimmo, London.

Avery, W.C. 1890. Birds observed in Alabama, Part 2. The Sportsman’s Journal 34:607-608.

Baker, R.H. 1956. Remarks on the former distribution of animals in eastern Texas. Texas Journal of Science 8:356-359.

Barde, F.S. 1912. Field, forest, and stream in Oklahoma. 1912 Annual Report to the State Game and Fish Warden.

Barton, B.S. 1799. Fragments of the natural history of Pennsylvania. Way & Groff, Philadelphia.

Bartram, W. 1958. The Travels of William Bartram. Yale University Press, New Haven.

Bartsch, P. 1895. Birds extinct in Iowa and those becoming so. Iowa Ornithologist 2:1-3.

Beckham, C.W. 1887. Additions to the avi-fauna of Bayou Sara, LA. The Auk 4:299-306.

Bendire, C.E. 1985. Life Histories of North American birds. U.S. National Special Bulletin No. 2 Smithsonian Contributions to Knowledge Vol. 32. Washington D.C.

Bent, A.C. 1940. Life histories of North American cuckoos, goat-suckers, hummingbirds and their allies. U.S. National Museum Bulletin 176. Washington D.C.

Bergtold, W.H. 1927. The Carolina paroquet in western New York. The Auk 44:252.

Bernhard, K. 1828. Travels through North America during the Years 1825 and 1826. Carey, Lea & Carey, Philadelphia.

Berthoud, E.L. 1887. Birds, their geological history, migration and uses. Transcript Press, Golden.

Beyer, M. and Koch, L. 1841. Amerikanische reisen. Immanuel Muller, Leipzig.

Bishop, N.H. 1879. Four months in a sneak-box. Lee & Shepard, Boston.

Blane, W.N. 1824. An excursion through the United States and Canada during the years 1822-23. Baldwin, Cradock & Joy, London.

Brasher, R. 1962. Birds and trees of North America. Rowman & Littlefield, New York.

Brewster, W. 1881. With the birds on a Florida river. Bulletin of the Nuttall Ornithological Club 6:38-44.

Brewster, W. 1889. Nesting habits of the parakeet (*Conurus carolinensis*). The Auk 6:336-337.

Bricknell, J. 1911. The Natural History of North Carolina. North Carolina State Library, Raleigh.

Brown, A. 1890. Genesis of the United States. Houghton Mifflin, Boston.

Bruff, J.G. 1849. Gold rush; the journals drawings, and other papers of J. Goldborough Bruff. Columbia University Press, New York.

Bryant, W.S. 1952. Henry Bryant, M.D., 1820-1867: a biography. Craftsman Press, New York.

Butler, A.W. 1892. Notes on the range and habits of the Carolina parrakeet. The Auk 9:49-56.

Butler, A.W. 1931. Some bird records from Florida. The Auk 48:436-439.

Carter, L. 1965. The diary of Colonel Landon Carter of Sabine Hall, 1752-1778. Virginia Historical Society, Richmond.

Carver, J. 1976. The Journals of Jonathan Carver and Related Documents, 1766-1770 (ed. J. Parker). Minnesota Historical Society Press, St. Paul.

Case, Z. 1951. The migration of Zophar Case from Cleveland to Vandalia, 1829-30. Northwest Ohio Quarterly 24:83-91.

Catlin, G. 1857. Letters and notes on the manners, customs, and conditions of the North American Indians. Willis. P. Hazard, Philadelphia.

Chapman, F.M. 1890. Remarks on a trip to Brevard County, Florida in the spring of 1889. Proceedings of the Linnaean Society of New York 2:2.

Chapman, F.M. 1890. Notes on the Carolina paroquet (*Conurus* *carolinensis*) in Florida. Proceedings of the Linnaean Society of New York 2:4-6.

Charlevoix, P.F.X. 1923. Journal of a Voyage to North America. Caxton Club, Chicago.

Childs, J.L. 1906. Eggs of the Carolina paroquet (*Conurus carolinensis*). The Warbler 2:65.

Christy, B.H. 1936. Kirtland marginalia. Cardinal. 4:77-89.

Clark, W. 1964. The Field Notes of Captain William Clark 1803 – 1805. Yale University Press, New Haven.

Coale, H.K. 1894. Ornithological notes on a flying trip through Kansas, New Mexica, Arizona and Texas. The Auk 11:215-222.

Cohen, M.M. 1836. Notices of Florida and the campaigns. Charleston.

Collot, G.H.V. 1924. A journey in North America, containing a survey of the counties watered by the Mississippi, Ohio, Missouri, and other affluing rivers. O. Lange Firenze, New York.

Cooke, W.W. 1888. Report on the migration in the Mississippi Valley in the years 1884 and 1885. U.S. Dept. Agriculture Division. Econ. Ornithology, Washington D.C.

Cooke, W.W. 1914. Some winter birds of Oklahoma. The Auk. 31:473-493.

Coues, E. 1877. Western range of *Conurus carolinensis*. Bulletin of the Nuttall Ornithological Club 2:50.

Cox, S.C. 1860. Recollections of the early settlement of the Wabash valley. Courier Steam Book & Job Printing House, Lafayette.

Cresswell, N. 1925. The Journal of Nicholas Cresswell 1774-1777. Jonathan Cape, London.

Cuming, F. 1810. Sketches of a tour to the western country, through the states of Ohio and Kentucky. Cramer, Spear & Eichbaum, Pittsburgh.

Davie, O. 1898. Nests and eggs of North American birds. 5^th^ Ed. Langdon Press, Columbus.

Davis, E.C. 1887. The Carolina parrot in northern Texas. Ornithology and Oology 12:62.

Davis, J. 1909. Travels of Four Years and a Half in the United States of America. Holt, New York.

Duck, L.G. and J.B. Fletcher. 1945. A survey of the game and furbearing animals of Oklahoma. Southwestern Stationary & Bank Supply, Ponca City.

Duden, G. 1829. Bericht uber eine Reise nach den westlichen Staaten Nordamerika’s und einer mehrjahrigen Aufenthalt am Missouri (in den Jahren 1824, 25, 26 und 1827). Sam Lucas, Elberfeld.

Du Ru, P. and Butler, R.L. 1934. Journal of Paul du Ru (February 1 to May 8, 1700), missionary priest of Louisiana. Caxton Club, Chicago, IL.

Eaton, W.F. 1936. Former occurrence of Carolina Paroquet in New Jersey. The Auk. 53:82.

Edward, D.B. 1836. The history of Texas. J.A. James and Co., Cincinnati, OH.

Emory, W.H. 1848. Notes of a military reconnaissance, from Fort Leavenworth, in Missouri, to San Diego, in California. U.S. Senate Ex. Doc. 7, 30th Congress, 1st Session, Washington D.C.

Evans, E. 1819. A pedestrious tour, of four thousand miles through the western states and territories. Joseph C. Spead, Concord.

Fables, D., Jr. 1955. Annotated list of New Jersey birds. Urner Ornithological Club.

Featherstonhaugh, G.W. 1835. Geological report of an examination made in 1834, of the elevated country between the Missouri and the Red Rivers. U.S. 23^rd^ Cong. 2^nd^ Sess. House Exec. Doc. 151:1-97.

Featherstonhaugh, G.W. 1844. Excursion through the Slave States, from Washington on the Potomac to the frontier of Mexico. Harper, New York.

Ferris, W.A. 1940. Life in the Rocky Mountains 1830-1835. Rocky Mountain Book Shop, Salt Lake City.

Fleming, W. 1916. Col. William Fleming's journal in Kentucky from Nov. 10, 1779 to May 27, 1780. Macmillam, New York.

Flint, T. 1826. Recollections of the last ten years. Cummings, Hilliard & Co., Boston.

Force, P. 1846. Tracts and other papers relating principally to the origin, settlement, and progress of the colonies of North America. Peter Force, Washington D.C.

Fordham, E.P. 1906. Personal narrative of travels in Virginia, Maryland, Ohio, Indiana, Kentucky. A.H. Clark, Cleveland.

Foreman, C.T. 1929. A Cherokee pioneer, Ella Floora Coodey Robinson. Chronicles of Oklahoma 7:363-374.

French, B.F. 1846. Historical Collections of Louisiana. Wiley & Putnam, New York.

Gale, J. 1969. The Missouri expedition 1818 – 1820 (R.L. Nichols, ed.). University of Oklahoma Press, Norman.

Ganier, A.F. A distributional list of the birds of Tennessee. Tennessee Avifauna. 1. (1933)

Genoways, H.H.; Ratcliffe, B. 2008. Engineer Cantonment, Missouri Territory, 1819-1820: America's First Biodiversity Inventory. Great Plains Research 8:3-31.

Gordon, T. 1909. Ivory-billed woodpecker. Forest and Stream 37:64.

Goss, N. S. 1886. A revised catalogue of the birds of Kansas. Kansas Publication House, Topeka.

Gosse, P.H. 1859. Letters from Alabama, Chiefly Relating to Natural History. Morgan & Chase, London.

Gratz, S. and T. Rodney. 1919. Pennsylvania Magazine of History & Biography 43:117-142.

Greene, M. 1856. The Kanzas region: forest, prairie, desert, mountain, vale, and river. Fowler & Wells, New York.

Guilday, J.E. 1971. Biological and archaeological analysis of bones from a 17^th^ century Indian village (46 PU 31), Putnam County, West Virginia. West Virginia Geological and Economic Survey, Report on Archeological Investigations No. 4.

Hahn, P. 1963. Where is that vanished bird? University of Toronto Press, Toronto.

Hall, J. 1828. Letters from the West. Henry Colburn, London.

Hamor, R. 1906. A true discourse of the present estate of Virginia. In: Hakluytus posthumus or Purchas his pilgrimes. Jas. LacLehose & Sons, Glasgow.

Hariot, T. 1588. A briefe and true report of the new found land of Virginia. London.

Harper, L. 1857. Preliminary report on the geology and agriculture of the state of Mississippi. State Printer, Jackson.

Harris, E. 1951. Up the Missouri with Audubon; the journal of Edward Harris. University Oklahoma Press, Norman.

Harris, H. 1919. Birds of the Kansas City region. Trans. Academy Sciences St. Louis. 23:1-371.

Harris, W.T. 1821. Remarks made during a tour through the United States of America, in the years 1817, 1818, and 1819. Sherwood, Neely & Jones, London.

Hasbrouck, E.M. 1891. The Carolina Paroquet (*Conurus carolinensis*). The Auk 8:369-379.

Hastings, R.W. 2009. The lakes of Pontchartrain: their history and environments. University Press of Mississippi, Jackson.

Hay, O.P. 1882. A list of birds from the lower Mississippi valley, observed during the summer of 1881, with brief notes. Bulletin of the Nuttall Ornithological Club 7:89-94.

Heartman, C.F. 1941. An immigrant of a hundred years ago. The Book Farm, Hattiesburg.

Hecklewelder, J. 1888. Narrative of John Hecklewelder’s journey to the Wabash in 1792. Pennsylvania Magazine of History and Biography 12:165-184.

Henshaw, H.W. 1919. Autobiographical notes. The Condor 21:169.

Herrick, F.H. 1917. Audubon, the Naturalist, a History of his Life and Time. Appleton-Century, New York.

Hertzel, A.X. 2004. A Minnesota Record of the Carolina Parakeet. The Loon. 76:175-181.

Hildreth, S.P. 1826. Facts relating to certain parts of the state of Ohio. American Journal of Science 10:1-8; 152-162; 319-331.

Hildreth, S. 1842. History of an early voyage on the Ohio and Mississippi rivers. American Pioneer. 1:89-105, 128-145.

Hilton, W. 1664. A relation of a discovery lately made on the coast of Florida. London.

Hoffman, C.F. 1835. A winter in the West, by a New Yorker. Harper, New York.

Hopkins, G.T. 1862. A mission to the Indians, from the Indian Committee of Baltimore Meeting, to Fort Wayne in 1804. T. Elwod Zell, Philadelphia.

Howe, H. 1847. Historical collections of Ohio. Author, Cincinnati.

Howell, A.H. 1911. Birds of Arkansas. U.S. Biological Survey Bulletin 38:1-100.

Howell, A.H. 1932. Florida bird life. Florida Department of Game and Fresh Water Fish, Tallahasee.

Hoy, P.R. 1865. Journal of an exploration of western Missouri in 1854. Smithsonian Institution Annual Report, Washington D.C.

Hume, E.E. 1942. Ornithologists of the United States Army Medical Corps. Johns Hopkins Press, Baltimore.

Irving, J.T., Jr. 1955. Indian sketches, taken during an expedition to the Pawnee tribes. University Oklahoma Press, Norman.

Irving, W. 1944. The western journals of Washington Irving. University Oklahoma Press, Norman.

James, E. 1823. Account of an expedition from Pittsburgh to the Rocky Mountains, performed in the years 1819, 1820. Longman, Hurst, Rees, Orme & Brown, London, UK.

Jefferson, T. 1894. The writings of Thomas Jefferson, Vol. 3 (P.L. Ford, ed.). Putnam’s. New York.

Jenks, J.W.P. 1878. Hunting in Florida in 1874. Forest and Stream 29:344.

Jennings, J. 1907. Journal from Fort Pitt to Fort Chartres in the Illinois country, March-April, 1766. Pennsylvania Magazine of History & Biography 31:145-156.

Johnston, D.W. 2002. Additional 16th Century bird reports From Florida. Florida Field Naturalist 30:1-8.

Johnston, E. The diary of Eliza (Ms. Albert Sidney) Johnston; the Second Cavalry come to Texas. Southwestern History Quarterly 60:463-500.

Jones, D. 1865. A journal of two visits made to some nations of Indians on the west side of the River Ohio In the years 1772 & 1773. Sabin Prints, New York.

Kirtland, J.P. 1838. Report on the zoology of Ohio. Second Annual Report of the Geological Survey of the State of Ohio. 157-200.

Koch, L. 1841. “Dampfboot-Reise nach St. Louis am Mississippi” and “Reisen und Aufenthalt in Missouri.” In Amerikanische Reisen (M. Beyer and L. Koch, eds.). Immanuel Muller, Leipzig.

Koch, A. 1888. Zwei Monate in West-Florida. Ornithologischer Verein in Wein, Mittheilungen 12:1-4, 25-26.

Kumlien L. and N. Hollister. 1903. The birds of Wisconsin. Bulletin Wisonsin Natural History Society 3:1-143.

Langdon, F.W. 1881. Field notes on Louisiana birds. Journal of Cincinnati Society Natural History 4:145-155.

Langdon, F.W. 1887. A catalogue of the birds of the vicinity of Cincinnati, with notes. Naturalists' Agency, Salem.

Latrobe, C.J. 1835. The Rambler in North America. Harper, New York.

Laudonniere, R. 1602. A note of such commodities as are found in Florida next adjoining to the South part of Virginia, taken out of the description of the said countrey, written by Mounsieur Rene Laudonniere, who inhabited there two Sommers and one winter *in* A Breife and true Relation of the Discoverie of the North Part of Virginia (J. Brereton, ed). London.

Laurent, P. 1906. Bird notes from a Florida porch. Bird-Lore 8:67.

Lawrie, A. 1944. Lawries's trip to northeast Texas, 1854-1855. South-western History Quarterly 48:238-253.

Lewis, M. and W. Clark. 1904. Original journals of the Lewis and Clark expedition, 1804-1806. Dodd, Mead & Co., New York.

Libhart, J.J. 1869. Ornithology *in* an Authentic History of Lancaster County (J.I. Momber ed.). J. E. Barr & Co., Lancaster.

Loskiel, G.H. 1794. History of the mission of the United Bretheren among the Indians of North America. Bretheren’s Society & John Stockdale, London.

Lyman, H.M. and Elmore, S.E. 1857. The Florida expedition. Williams College Quarterly 4:293-328.

Macbride, T.H. 1928. In cabins and sod-houses. State History Society of Iowa, Iowa City.

Macdonald, D. 1942. The diaries of Donald Macdonald 1824-1826. Indiana History Society Publication 14:143-379.

Mason, R.L. 1915. Narrative of Richard Lee Mason in the Pioneer West, 1819. Charles F. Heartman, New York.

Maynard, C. J. 1881. The birds of eastern North America. C.J. Maynard Company, Newtonville.

Maynard, C.J. 1928. Adventures of a naturalist in Florida from 1866-1900. Florida Naturalist 2:50.

McAtee, W.L. 1905. Ecological notes on the birds occurring within the radius of five miles of the Indiana University campus. Proceedings of the Indiana Academy of Sciences 1904:65-202.

McCrary, J.M. 1891. The Carolina paroquet in Florida. Forest and Stream 37:183.

McKinley, D. 1960. The Carolina Parakeet in Pioneer Missouri. The Wilson Bulletin 72:274-287.

McKinley, D. 1964. History of the Carolina Parakeet in its Southwestern Range. The Wilson Bulletin 76:68-93.

McKinley, D. 1965. The Carolina Parakeet in the Upper Missouri and Mississippi river valleys. Auk 82:215-226.

McKinley, D. 1976. The Carolina Parakeet in Indiana. Indiana Audubon Quarterly 54:97-107.

McKinley, D. 1977. Records of the Carolina Parakeet in Ohio. Ohio Journal of Science 77:3.

McKinley, D. 1977. The Carolina Parakeet in Georgia: a review of reports. The Oriole 42:21-25.

McKinley, D. 1978. The Carolina Parakeet in the Virginias: a review. The Raven 49:3-10.

McKinley, D. 1978. The Carolina Parakeet in Alabama, a History. EOS (Alabama Museum Natural History) 1:7.

McKinley, D. 1978. The Carolina Parakeet in Illinois: a recapitulation. Indiana Audubon Quarterly. 56:53-68.

McKinley, D. 1978. The Carolina parakeet in the west: additional references. The Nebraska Bird Review 46:3-7.

McKinley, D. 1979. A Review of the Carolina Parakeet in Tennessee. The Migrant 50:1-6.

McKinley, D. 1979. Historical review of the Carolina parakeet in the Carolinas. Brimleyana. 81-89.

McKinley, D. 1979. History of the Carolina parakeet in Pennsylvania, New Jersey, Delaware, Maryland and the District of Columbia. Maryland Birdlife 35:3-10.

McKinley, D. 1979. The Carolina Parakeet in Kentucky. The Indiana Audubon Quarterly 57:187-195.

McKinley, D. 1981. The Carolina Parakeet in Mississippi: A Historical Review. The Mississippi Kite. 11:2-6.

McKinley, D. 1985. The Carolina Parakeet in New York and Ontario: A Review and a Footnote. The Kingbird. 167-172.

McKinley, D. and J. W. Hardy. 1985. The Carolina Parakeet in Florida. Florida Ornithological Society, Gainesville.

McKinley, D. and D. James. 1984. A Summary Account of the Carolina Parakeet in Arkansas. Arkansas Academy of Science Proceedings 38:64-67.

Meredith, H. 1922. An Account of the Cape Fear Country 1731. Charles F. Heartman, Perth Amboy.

Merriam, C.H. 1874. Ornithological notes from the South II. American Naturalist 8:85-89.

Merriam, C.H. 1892. The Carolina Paroquet (*Conurus carolinensis*) in Missouri. The Auk 9:301.

Michaux, A. 1904. Journal of travels into Kentucky July 15, 1793-April 11, 1796. A.H. Clark, Cleveland.

Mills, R. 1826. Statistics of South Carolina, including a View of its Natural, Civil, and Military History, General and Particular. Hulbert & Lloyd, Charleston.

Miranda, F. D. 1963. The diary of Francisco de Miranda; tour of the United States 1783-1784. University of Oklahoma Press, Norman.

Mitchell, H.L. 1869. Note on the parakeets at Tampa. Florida Peninsular (27 Feb ed.).

Mittleberger, G. 1960. Gottlieb Mittleberger’s Journey to Pennsylvania in the Year 1750. Belknap Press, Harvard University, Cambridge.

Mollhausen, B. 1858. Diary of a journey from the Mississippi to the coasts of the Pacific with the United States government expedition. Longmans & Roberts, London.

Mooney, C.C. 1961. From Old Vincennes, 1815 (letter of Caleb Lownes). Indiana Magazine of History 57:141-154.

Moseley, E.L. 1904. Notes from Sandusky, Ohio. Wilson Bulletin 16:112.

Mulkearn, L. 1954. George Mercer papers relating to the Company of Virginia. University Pittsburgh Press, Pittsburgh.

Murphey, E.E. 1937. Observations on the bird life of the middle Savannah valley 1890-1937. Charleston Museum, Charleston.

Murray, A.M. 1857. Letters from the United States, Cuba and Canada. Putnam & Co., New York.

Murray, C.A. 1839. Travels in North American during the years 1834, 1835, and 1836. Harper & Bros., New York.

Nehrling, H. 1896. Our native birds of song and beauty. Brumder, Milwaukee.

Nice, M. M. 1931. The birds of Oklahoma. Publication University Oklahoma Biol. Survey 3:1-224.

North, A. 1923. Asahel North – biographic sketch and diary. Journal of the Illinois State Magazine of History 57:141-154.

Nutall, T. 1821. Journal of travels into the Arkansa territory, during the year 1819. Thos. W. Palmer, Philadelphia.

Nutall, T. 1840. A Manual of the Ornithology of the United States and of Canada - Land Birds Hilliard, Gray & Co., Boston.

“Ober.” 1874. Forest and Stream 2:162.

Oberholser, H.S. 1938. The Bird Life of Louisiana. Louisiana Department of Conservation. Baton Rouge.

Oberholser, H.C. 1974. The bird life of Texas. University of Texas Press, Austin.

Owen, W. 1906. Diary of William Owen from November 10, 1824 to April 20, 1825. Indiana History Society Publication 4:1-134.

Page, F.B. 1846. Prairiedom; rambles and scrambles in Texas or New Estremadura (2^nd^ ed.). Paine & Burgess, New York.

Peale, T.R. 1947. The journal of Titian Ramsay Peale, pioneer naturalist. Missouri History Review 41:147-163, 266-284.

Pierson, G.W. 1938. Tocqueville and Beaumont in America. Oxford University Press, New York.

Pindar, L.O. 1889. List of birds of Fulton County, Kentucky. The Auk 6:310-316.

Quaife, M.M. 1947. The western country in the 17th century; the memoirs of Lamothe Cadillac and Pierre Liette. Donnelley, Chicago.

Read, M.C. 1853. Catalogue of the birds of northern Ohio. Proceedings of the Academy of Natural Sciences Philadelphia 6:395-402.

“Red-Wing.” 1885. Rare Florida Birds. Forest and Stream 24:487.

Reeves, E. 1896. Extracts from the letter-books of Lt. Enos Reeves, of the Pennsylvania Line. Pennsylvania Magazine of History and Biography 20:302-314.

Rhoads, S.N. 1895. Contributions to the zoology of Tennessee, No. 2. Birds. Proceedings of the Academy of Natural Sciences Philadelphia. 47:463-501.

Ridgway, R. 1916. Birds of North and Middle America, Pt. 7. U.S. National Museum Bulletin 50:1-543.

Salley, A.S. 1911. Narratives of Early Carolina, 1650-1708. Scribners, New York.

Schoolcraft, H.R. 1819. A view of the lead mines of Missouri. C. Wiley & Co., New York.

Schoolcraft, H.R. 1825. Travels in the central portions of the Mississippi valley. Collins & Hannay, New York.

Schoolcraft, H.R. 1851. Personal memoirs of a residence of thirty years with the Indiana tribes of the American frontiers. Lippincott, Grambo, & Co., Philadelphia.

Scott, W.E.D. 1881. On birds observed in Sumter, Levy, and Hillsboro’ counties, Florida. Bulletin of the Nuttall Ornithological Club 6:14-21.

Scott, W.E.D. 1889. A summary of observations on the birds of the Gulf coast of Florida. The Auk 6:249-250.

Scott, W.E.D. 1903. The story of a bird lover. Outlook Co., New York.

Scoville, S., Jr. 1940. Ivory-bills and paroquets. Yale Review 29:556-564.

Shirreff, P. 1835. A tour through North America. Oliver & Boyd, Edinburgh.

Smith, H.M. and W. Palmer, 1888. Additions to the avifauna of Washington and vicinity. The Auk 5:147-148.

Snyder, N. F. 2004. The Carolina Parakeet: Glimpses of a vanished bird. Princeton University Press, Princeton.

Steiner, A. and de Schweinitz, F.C. 1927. Report of the journey of the Brethren Abraham Steiner and Frederick C. de Schweinitz to the Cherokees and the Cumberland settlements (1799). Watuga Press, Johnson City.

Stresemann, E. 1954. Augestorbene und austerbende volgelarten, vertreten im zoologische Museum zu Berlin. Mitteilungen aus den Zoologischi Museum Berlin 30:38-53.

Stone, W. 1894. The birds of eastern Pennsylvania and New Jersey. Delaware Valley Ornithological Club, Philadelphia.

Thomas, D. 1819. Travels through the Western Country in the summer of 1816. David Rumsey, Auburn, NY.

Thompson, C. and M.C. Ely. 1989. Birds in Kansas, Vol. 1. University Press of Kansas, Lawrence.

Tixier, V. 1940. Tixier's travels on the Osage prairies. University Oklahoma Press, Norman, OK.

Townsend, F.T. 1875. Wild life in Florida. Hurst & Blackett, London.

Townsend, J.K. 1839. Narrative of a journey across the Rocky Mountains to the Columbia River. Perkins & Marvin, Philadelphia.

Trippe, T.M. 1873. Notes on the birds of southern Iowa. Proceedings Boston Society Natural History 15:229-242.

von Reck, P.F. 1846. An extract of the journals… the first transport of Salzburgers to Georgia. Peter Force's Tracts and Other Papers 4:1-37.

“W.” 1904. A cheap winter in Florida. Forest and Stream 63:442.

Warren, G.K. 1875. Preliminary report on explorations in Nebraska and Dakota, in the years 1855-56-57. Government Printing Office, Washington, D.C.

Wayne, A.T. 1895. Notes on the birds of the Wacissa and Aucilla river regions of Florida. The Auk 12:362-367.

Wheaton, J.M. 1882. Report on the birds of Ohio. Report of the Geological Survey of Ohio 4:187-628.

Widmann, O. 1907. A preliminary catalog of the birds of Missouri. Transactions of the Academy of Sciences St. Louis 17:1-288.

Wied-Neuwied, M.A.P. 1841. Reise in das innere Nord-America in den Jahren 1832 bis 1834. J. Hoelscher, Coblenz.

Wied-Neuwied, M.A.P. 1857. Ueber den Papagei von Nord America. *Psittacus carolinensis*. Lin. Journal fur Ornithologie 5:97-105.

Wied-Neuwied, M.A.P. 1906. Travels in the interior of North America (H.E. Lloyd, trans.) Vols. 22-25 *in* Early Western Travels (R.G. Thwaites, ed.). A.H. Clark Co., Cleveland.

Wilhelm, P. 1941. First journey to North America in the years 1822 to 1824. South Dakota History Collection 19:7-47.

Wilson, A. 1811. American ornithology; or, the natural history of the birds of the United States, Vol. 3. Bradford & Inskeep, Philadelphia.

Wilson, A. 1828. American ornithology with a sketch of the author's life by George Ord. Harrison Hall, Philadelphia.

Woods, J. 1822. Two years’ residence in the settlement on the English prairie, in Illinois country. Longman, Hurst, Rees, Orme, & Brown, London.

Wright, A.H. 1912. Early records of the Carolina Parakeet. The Auk. 29:343-363.

Wyman, J. 1899. A geographical sketch of the Alabama Territory. Transactions of the Alabama History Society 3:107-127.

**
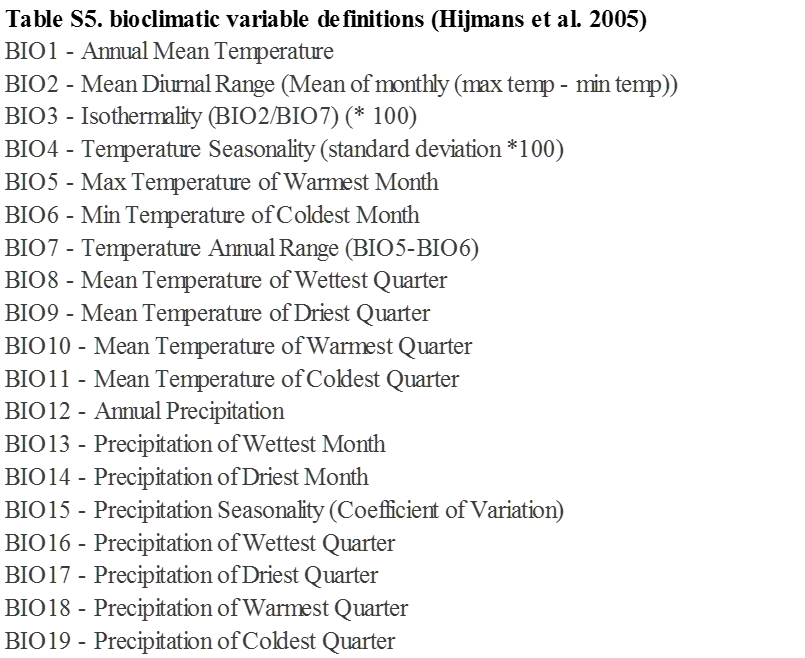
**

**SI Figures**

**
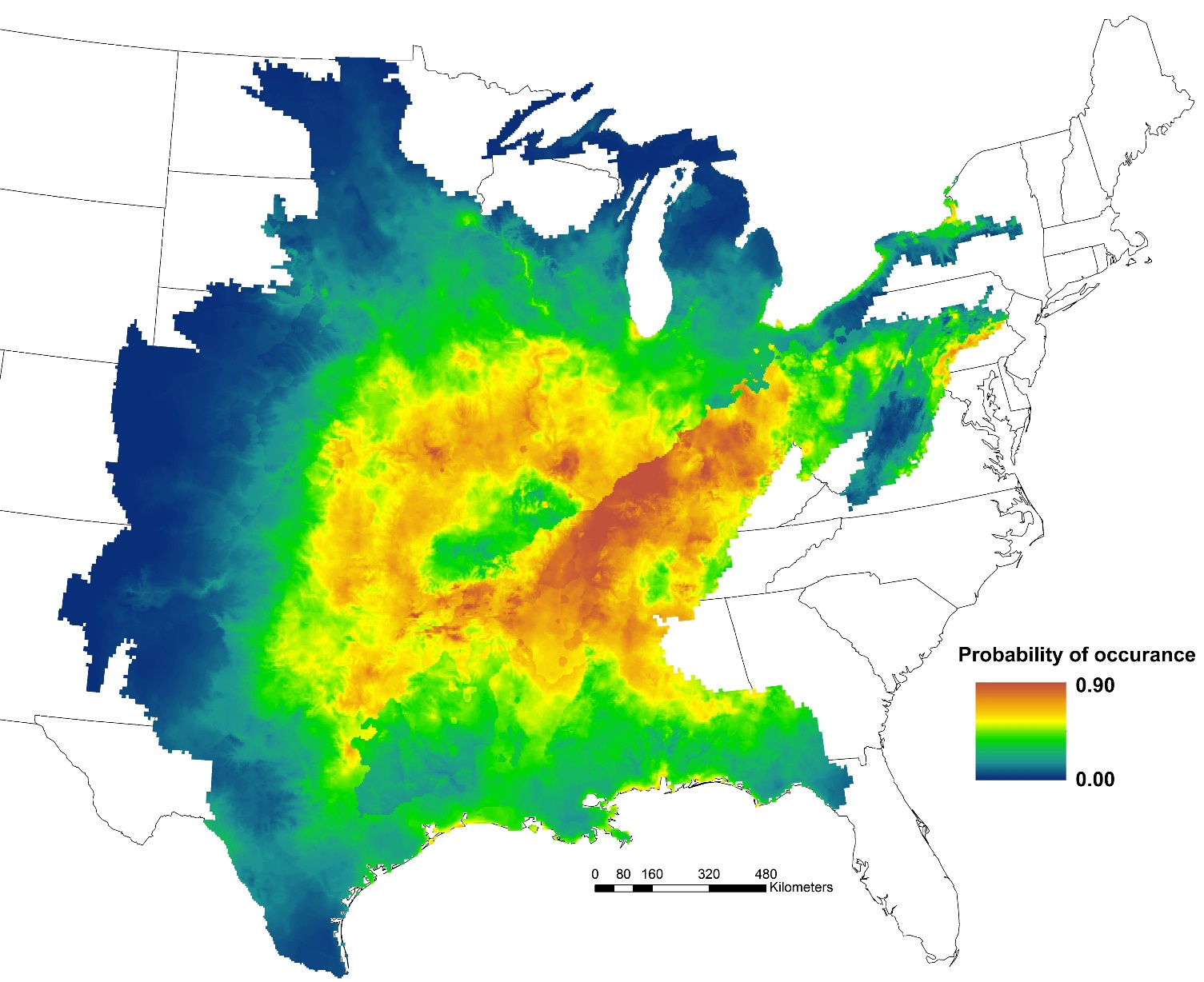
**

**Figure S1.** Full MaxEnt SDM prediction map for *C. c. ludovicianus* observations: AUC = 0.790. Red areas are areas with a relatively high probability and blue are areas with a low probability of occurrence. Map projection: North America Albers Equal Area Conic.


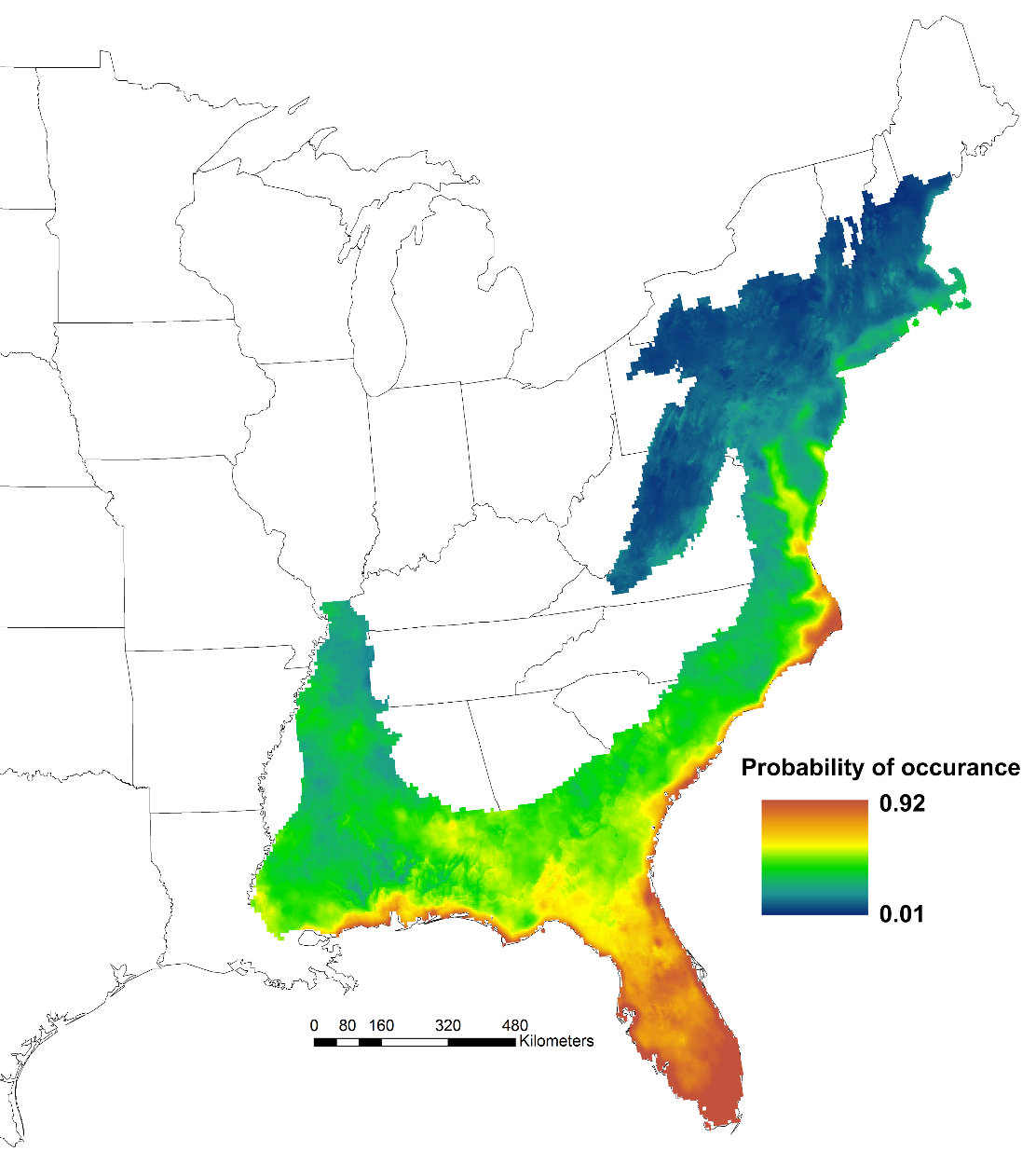


**S2**. Full MaxEnt SDM prediction map for all *C. c. carolinensis* observations: AUC = 0.814. Red areas are areas with a relatively high probability and blue are areas with a low probability of occurrence. Map projection: North America Albers Equal Area Conic.

**
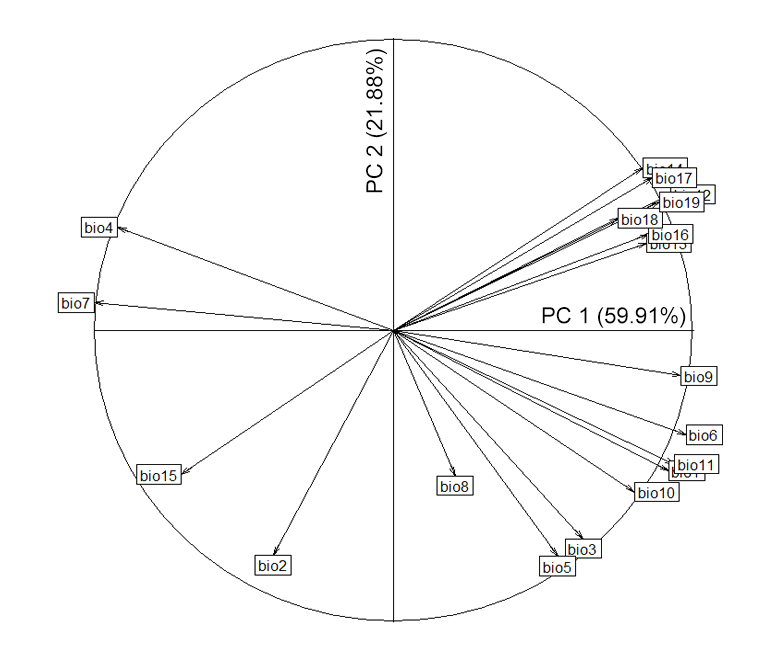
**

**S3.** Results of the contribution of the bioclimatic variables to the axes of the “within environment” PCA niche equivalency analysis comparing *C. c. ludovicianus* and *C. c. carolinensis*. See Table S5 for a description of the bioclimatic variables.

**
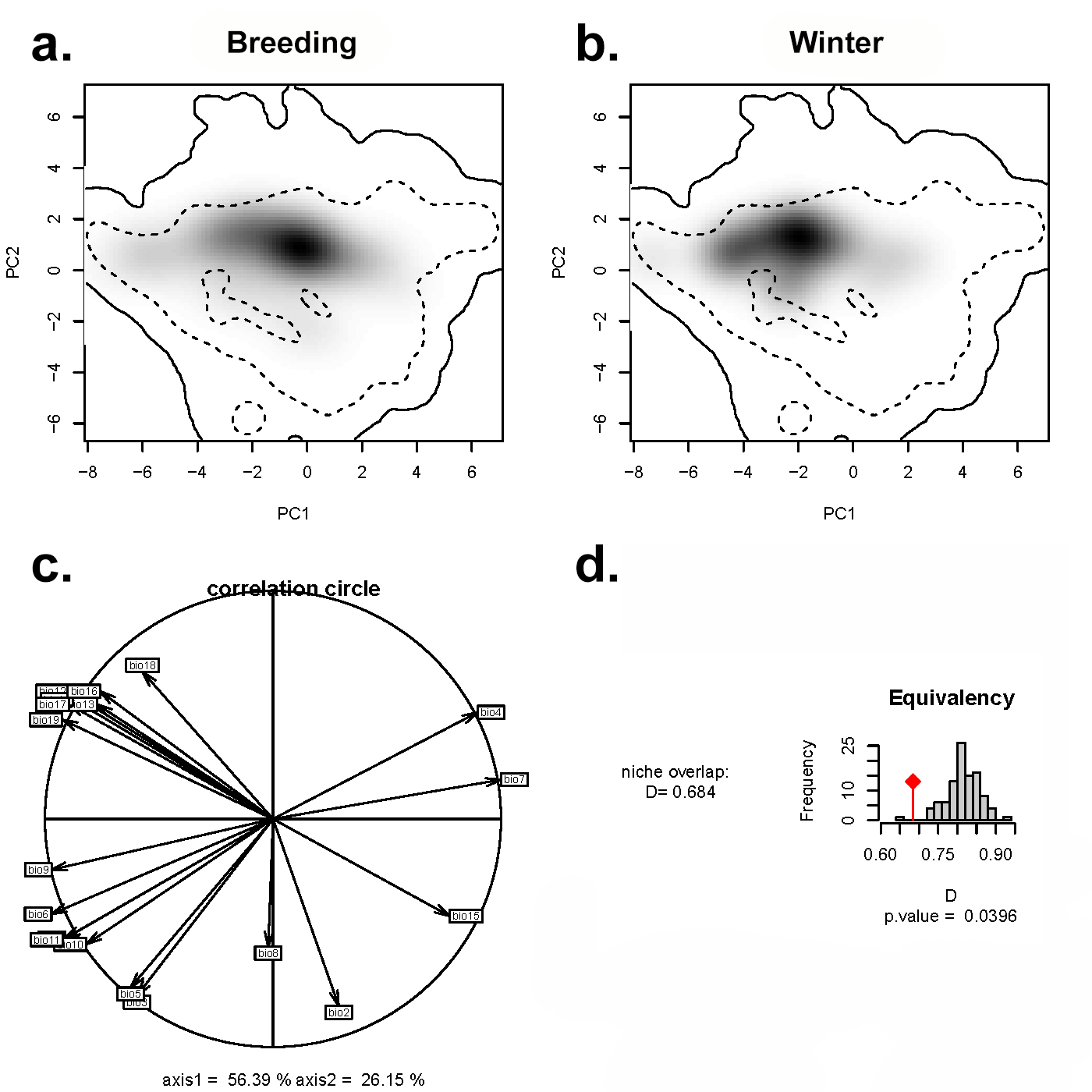
**

**S4**. Full results of the “within environment” PCA niche equivalency analyses between using all 19 bioclimatic variables breeding (a) and winter (b) seasons for *C. c. ludovicianus*. The shading reflects the density of occurrences of each subspecies per cell (i.e. darker cells have a higher density), the solid line within the PCA space represent 100% of the available climate space and the dotted lines represent 50% of the available climate space. (c) The contribution of the bioclimatic variables to the axes of the PCA analysis. See Table S5 for a description of the bioclimatic variables. (d) The red flag is the niche overlap (D = 0.684) and the histogram represents the simulated overlap between the two subspecies. The empirical overlap is significantly different from the simulated overlap (p = 0.0396).

**
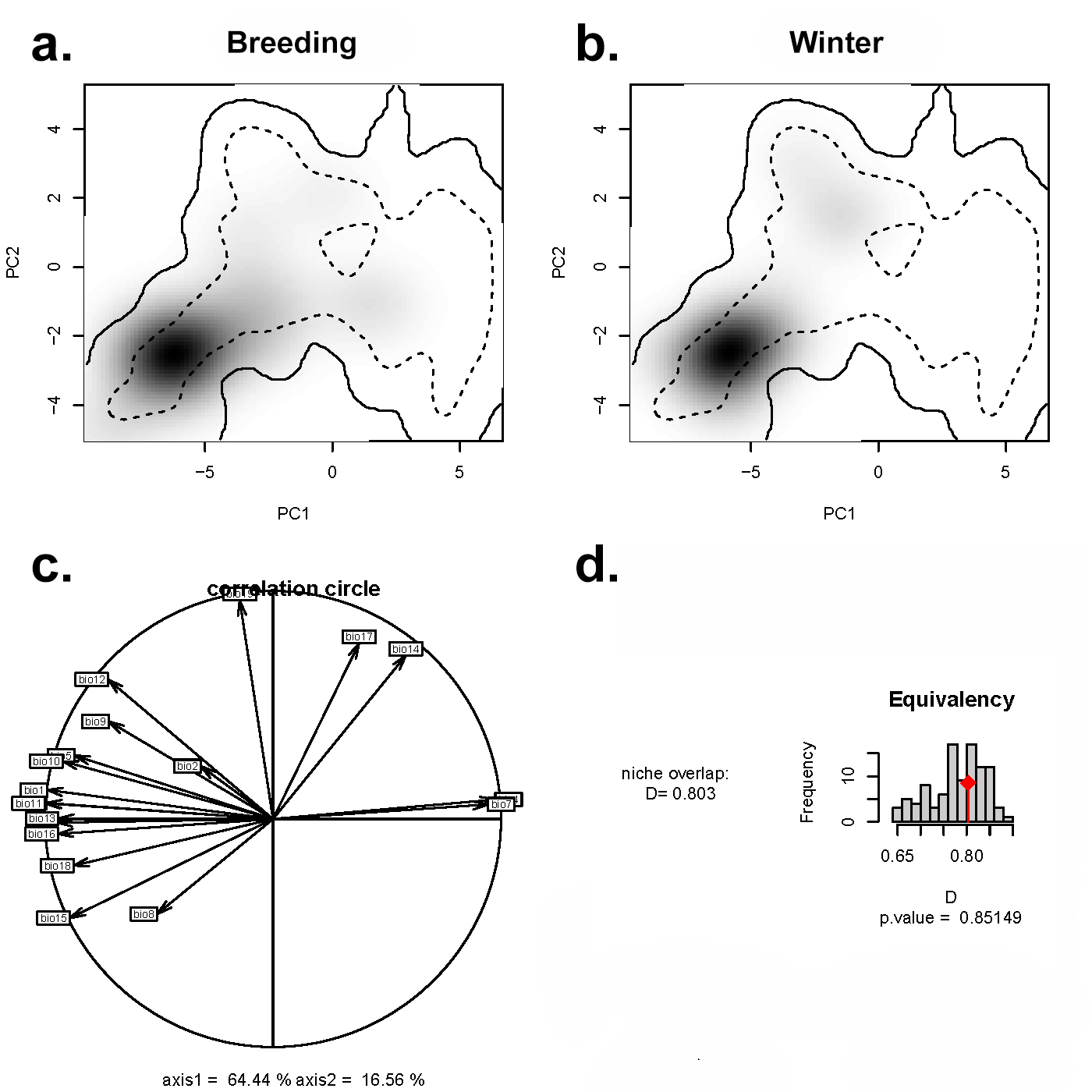
**

**S5**. Full results of the “within environment” PCA niche equivalency analyses between using all 19 bioclimatic variables breeding (a) and winter (b) seasons for *C. c. carolinensis*. The shading reflects the density of occurrences of each subspecies per cell (i.e. darker cells have a higher density), the solid line within the PCA space represent 100% of the available climate space and the dotted lines represent 50% of the available climate space. (c) The contribution of the bioclimatic variables to the axes of the PCA analysis. See Table S5 for a description of the bioclimatic variables. (d) The red flag is the niche overlap (D = 0.803) and the histogram represents the simulated overlap between the two subspecies. The empirical overlap was not significantly different from the simulated overlap (p = 0.85149).

**
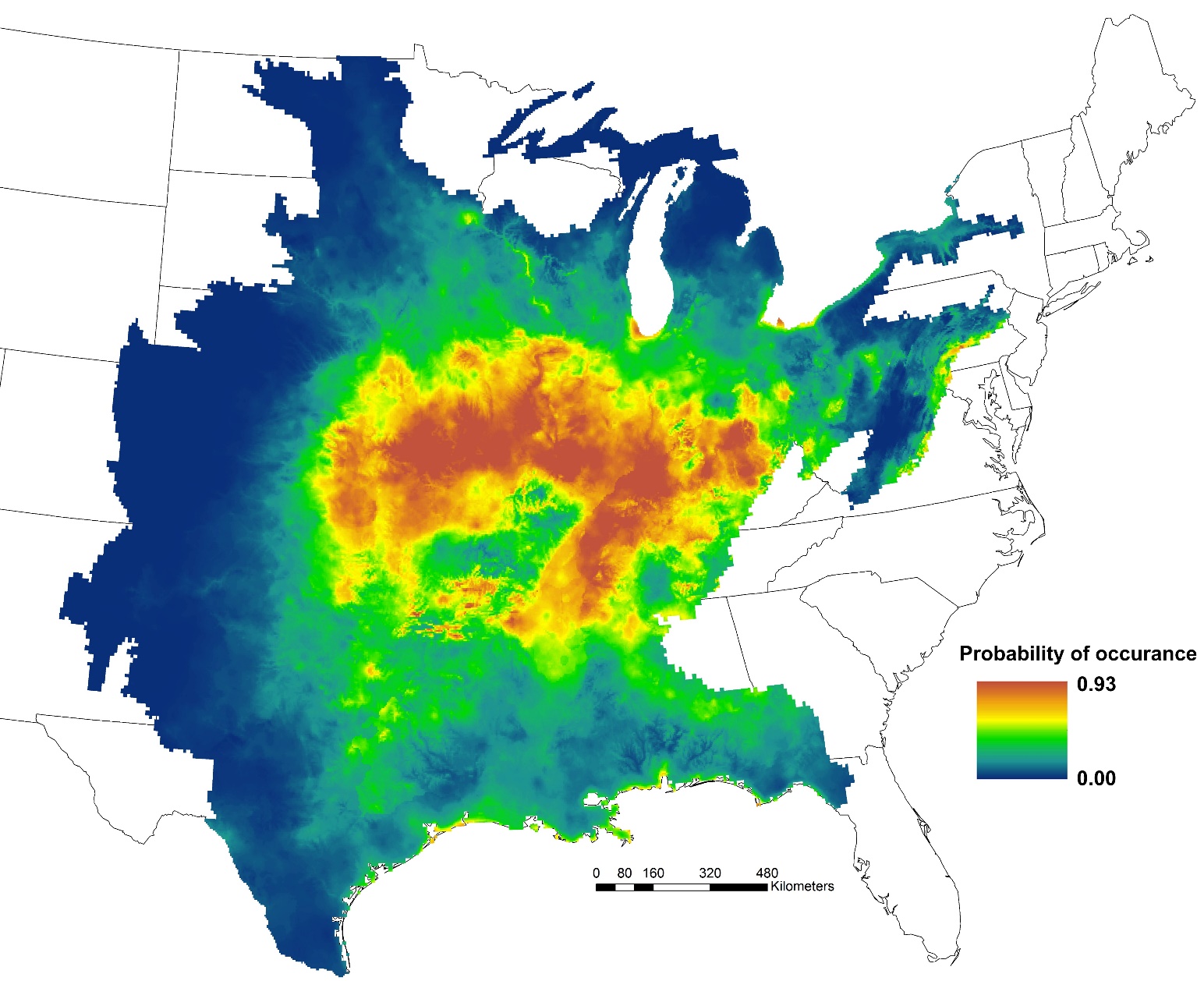
**

**S6**. Full MaxEnt SDM prediction map based on breeding season occurrences (March – August) of *C. c. ludovicianus*: AUC = 0.863. Red areas are areas with a relatively high probability and blue are areas with a low probability of occurrence. Map projection: North America Albers Equal Area Conic.

**
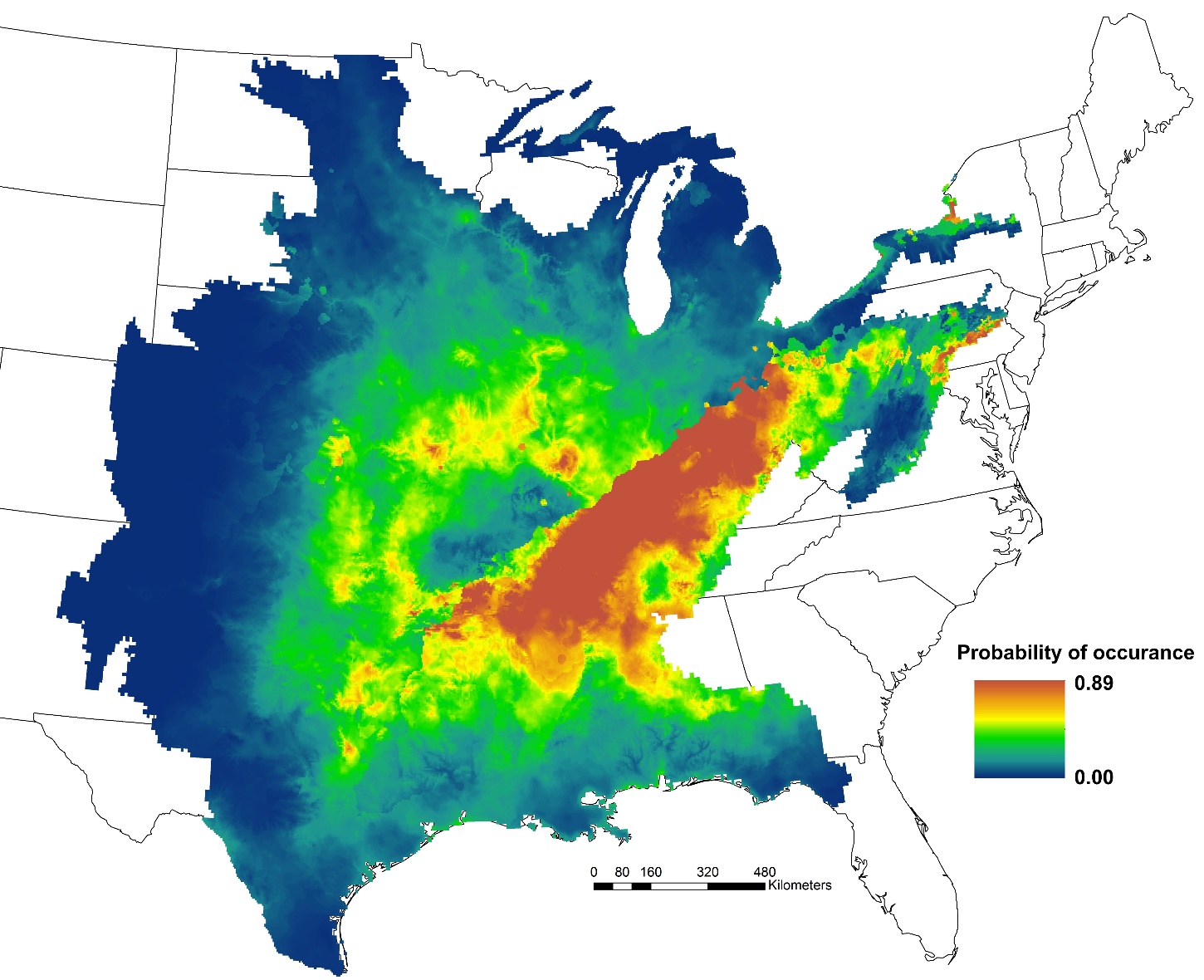
**

**S7.** Full MaxEnt SDM prediction map based on winter occurrences (December – February) *C. c. ludovicianus*: AUC = 0.885. Red areas are areas with a relatively high probability and blue are areas with a low probability of occurrence. Map projection: North America Albers Equal Area Conic.


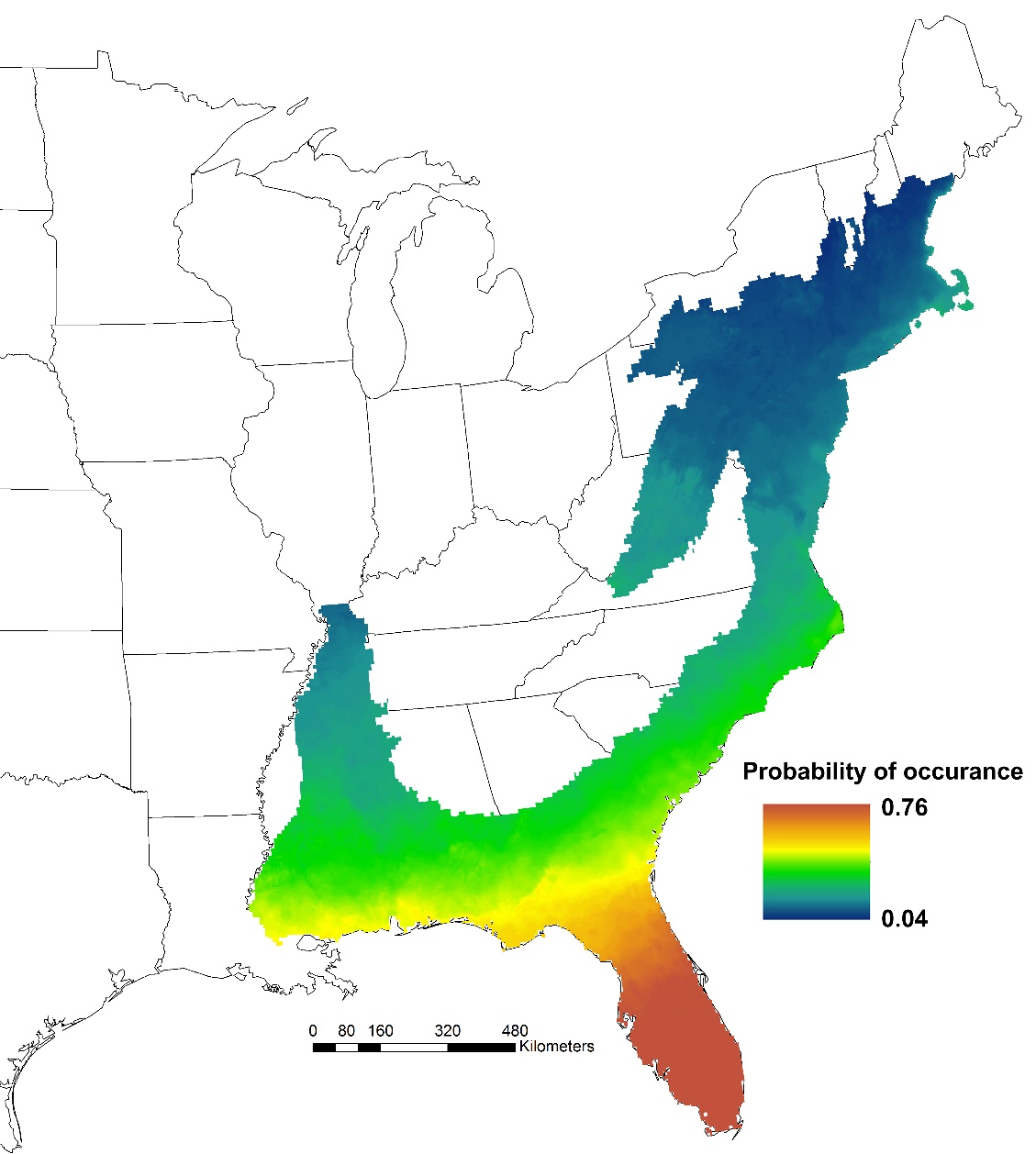


**S8.** Full MaxEnt SDM prediction map based on breeding season occurrences (March – August) of *C. c. carolinensis*: AUC = 0.845. Red areas are areas with a relatively high probability and blue are areas with a low probability of occurrence. Map projection: North America Albers Equal Area Conic.


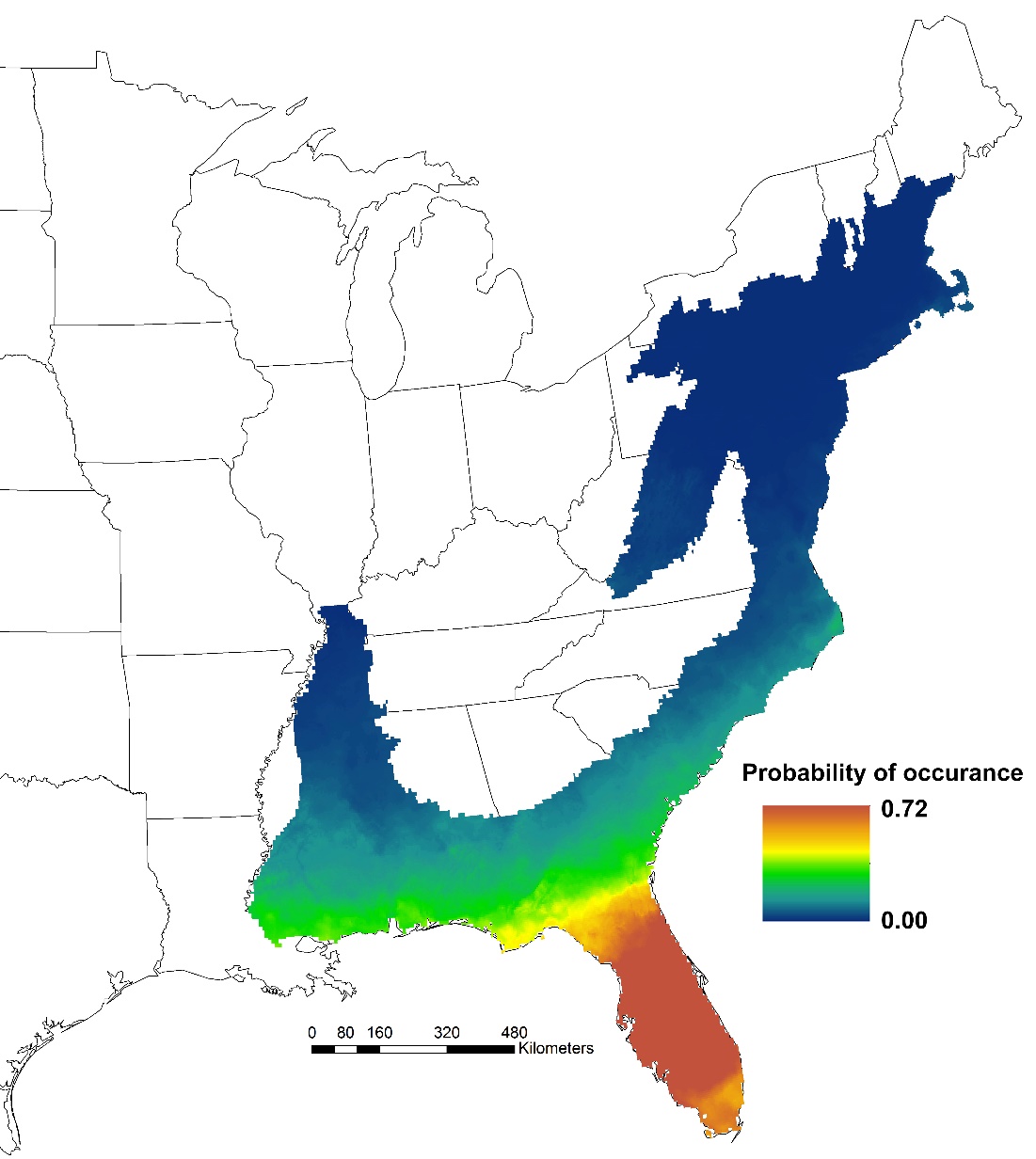


**S9.** Full MaxEnt SDM prediction map based on winter occurrences (December – February) *C. c. carolinensis*: AIC = 0.916. Red areas are areas with a relatively high probability and blue are areas with a low probability of occurrence. Map projection: North America Albers Equal Area Conic.
